# Dehydroandrographolide facilitates M2 macrophage polarization by downregulating DUSP3 to inhibit sepsis‐associated acute kidney injury

**DOI:** 10.1002/iid3.1249

**Published:** 2024-04-17

**Authors:** Yanyan Shao, Weihao Yu, Hailun Cai

**Affiliations:** ^1^ Department of Pediatrics The Second Affiliated Hospital of Wenzhou Medical University Wenzhou City China

**Keywords:** acute kidney injury, dehydroandrographolide, DUSP3, inflammation, M2 macrophage polarization

## Abstract

**Background:**

Sepsis is perceived as lethal tissue damage and significantly increases mortality in combination with acute kidney injury (AKI). M2 macrophages play important roles in the secretion of anti‐inflammatory and tissue repair mediators. We aimed to study the role of Dehydroandrographolide (Deh) in sepsis‐associated AKI in vitro and in vivo through lipopolysaccharide (LPS)‐induced macrophages model and cecal ligation and puncture‐induced AKI mice model, and to reveal the mechanism related to M2 macrophage polarization.

**Methods:**

Enzyme‐linked immunosorbent assay kits were used to assess the levels of inflammatory factors. Expression of markers related to M1 macrophages and M2 macrophages were analyzed. Additionally, dual specificity phosphatase 3 (DUSP3) expression was tested. Cell apoptosis was evaluated by flow cytometry analysis and terminal‐deoxynucleotidyl transferase‐mediated nick end labeling staining. Moreover, renal histological assessment was performed by using hematoxylin and eosin staining.

**Results:**

Deh reduced inflammation of THP‐1‐derived macrophages exposed to LPS. Besides, Deh induced the polarization of M1 macrophages to M2 and downregulated DUSP3 expression in THP‐1‐derived macrophages under LPS conditions. Further, DUSP3 overexpression reversed the impacts of Deh on the inflammation and M2 macrophages polarization of THP‐1‐derived macrophages stimulated by LPS. Additionally, human proximal tubular epithelial cells (HK‐2) in the condition medium from DUSP3‐overexpressed THP‐1‐derived macrophages treated with LPS and Deh displayed decreased viability and increased apoptosis and inflammation. The in vivo results suggested that Deh improved the renal function, ameliorated pathological injury, induced the polarization of M1 macrophages to M2, suppressed inflammation and apoptosis, and downregulated DUSP3 expression in sepsis‐induced mice.

**Conclusion:**

Deh facilitated M2 macrophage polarization by downregulating DUSP3 to inhibit septic AKI.

## Graphical abstract

Dehydroandrographolide exerts an alleviating effect on sepsis‐associated acute kidney injury, likely resulting from the promotion of M2 macrophage polarization by downregulating DUSP3.

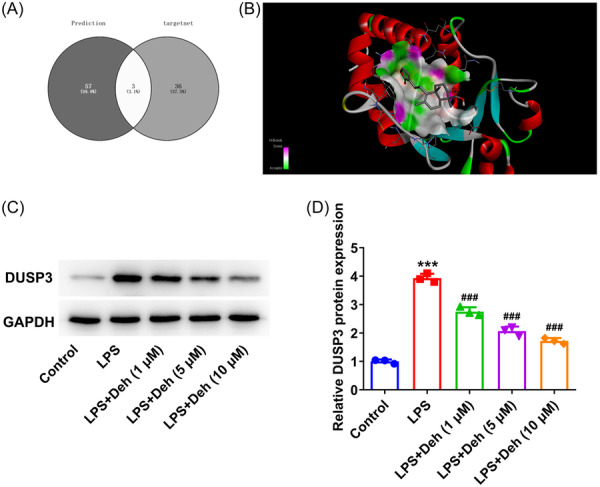

## 1 INTRODUCTION

Sepsis is a complex clinical syndrome initiated by a complicated systemic host response to infection, which has been defined as a severe, undeniable health threat by World Health Organization.^1^ It is estimated that ~19 million people worldwide suffer from sepsis each year and the in‐hospital mortality rate for patients with sepsis is about 15%–25%.^2^, ^3^ Acute kidney injury (AKI) is well documented as a grievous and prevalent complication of sepsis, and is commonly recognized as an independent influence factor for elevated lethality risk in sepsis patients, as the kidney is one of the most vulnerable target organs for sepsis.^4^ It is found that more than 50% of patients with severe sepsis develop the symptoms of AKI, and sepsis‐associated AKI increases the risk of developing chronic comorbidities and is closely associated with high mortality.^4^ AKI occurs in 17%–56% of critically ill children admitted to pediatric intensive care units worldwide and is associated with a 28‐day mortality rate of 11%.^5^ Despite significant advances in medicine and therapeutics, there is still no effective treatment for sepsis‐associated AKI in the clinic. Hence, it is imperative to develop novel therapeutic interventions for septic AKI.

An increasing number of studies have demonstrated that excessive inflammatory cascade reaction in septic AKI is one of the main causes of kidney damage.^6^, ^7^ Macrophages are an important component of innate and adaptive immunity, which play a crucial role in regulating inflammatory response in septic AKI.^8^, ^9^ Due to the plasticity and diversity of macrophages, macrophages can differentiate into two phenotypes, type M1 (classically activated) and type M2 (alternatively activated), which together maintain immune homeostasis within the body.^10^ M1 macrophages generate large amounts of pro‐inflammatory factors, including tumor necrosis factor‐α (TNF‐α), interleukin‐1β (IL‐1β), and interleukin‐6 (IL‐6), to destroy host pathogens, but prolonged inflammatory activity can lead to further tissue damage.^11^ In contrast, M2 macrophage suppresses pro‐inflammatory cytokine production and accelerates tissue repair and regeneration.^12^ Therefore, identifying the agents that facilitate the macrophages differentiation from M1 phenotype to M2 phenotype may provide therapeutic options for alleviating inflammation and promoting tissue repair during septic AKI.

*Andrographis paniculata* (Burm. f) Nees is an oriental herbal medicine that has been widely used for treating infection, inflammation, and hypertension due to its multiple beneficial pharmacological effects.^13^ In previous studies related to sepsis, andrographolide, an active component of *A. paniculata* (Burm. f) Nees, can decrease the mortality of mice with sepsis and inhibit the inflammatory response of mice with multimicrobial sepsis.^14^, ^15^ However, the effects of dehydroandrographolide (Deh), another main active constituent of *A. paniculata* (Burm. f) Nees, on sepsis remains to be elucidated. Particularly, Deh has been demonstrate to have significant anti‐inflammatory effects in vivo and in vitro. For instances, Xiong et al.^16^ has highlighted the important effect of Deh on enhancing innate immunity of intestinal tract. Deh shows the anti‐inflammatory activities in cholestatic liver injury, as evidenced by the reduced production of TNF‐α and IL‐6 in bile duct‐ligated mice or primary Kupffer cells treated with lipopolysaccharide (LPS).^17^ Combined with the above literature reports, we hypothesized that Deh may affect the occurrence of sepsis by inhibiting the inflammatory release of macrophages.

By using bioinformatics tools and molecular docking, we found that dual specificity phosphatase 3 (DUSP3), also called Vaccinia‐H1‐related phosphatase, could potentially been targeted by Deh. As a gene that strongly expressed in monocytes and macrophages, *DUSP3* plays a vital role as a positive regulator of the innate immune response.^18^ As reported, DUSP3 deficiency protects mice from endotoxemia and multimicrobially induced septic shock.^19^ In addition, DUSP3‐knockout mice display an increase in the proportion of M2‐type macrophages and a decrease in the expression of inflammatory factor TNF‐α.^18^ Of note, a previous study in the kidney has shown that DUSP3 silencing reduces kidney injury and inflammation after ischemia/reperfusion injury in mice.^20^

In this work, the effects of Deh on sepsis‐associated AKI were explored by using the LPS‐induced macrophages model and cecal ligation and puncture (CLP)‐induced AKI mice model. Further, the potential mechanism of Deh related to macrophage polarization and DUSP3 were investigated.

## 2 MATERIALS AND METHODS

### 2.1 Bioinformatics tools and molecular docking

The potential target genes of Deh were predicted by the Prediction (https://prediction.charite.de/) and Targetnet (http://targetnet.scbdd.com/calcnet/calc_text/) databases, and the results were exhibited using Venn diagram. The three‐dimensional structures of DUSP3 was obtained from PDB website (http://www.rcsb.org/) and saved in PDB format, which was then converted into “pdbqt” format using AutoDockTools 1.5.6. Subsequently, molecular docking was performed using Autodock (version 4.2) and the results were visualized with Pymol 2.5.2 (https://pymol.org/2/) software.

### 2.2 Cell culture and treatment

The human acute monocytic leukemia cell line THP‐1 and human proximal tubular epithelial cells (HK‐2) were purchased from BeNa Culture Collection. THP‐1 cells were grown in RPMI 1640 medium (HyClone) and HK‐2 cells were cultured in Dulbecco's modified Eagle medium/F12 medium (Life Technologies), both of which were supplemented with 10% fetal bovine serum (VivaCell). Cells were incubated in a CO_2_‐humidified incubator at 37°C.

In all experiments, THP‐1 monocytes cultured in six‐well plates were differentiated into macrophage‐like cells (M0 phenotype) by adding 100 nM phorbol‐12‐myristate‐13‐acetate into medium for 24 h incubation.^21^ Then, THP‐1‐derived macrophages were pretreated with various concentrations of Deh (1, 5, and 10 μM) for 1 h and then induced by LPS (1 μg/mL) for 24 h.^22^, ^23^ The condition medium (CM) from THP‐1‐derived macrophages were collected and used for treating HK‐2 cells for 24 h.

### 2.3 Transfection

To overexpress DUSP3 expression, THP‐1‐derived macrophages were transfected with DUSP3 plasmid (Ov‐DUSP3) using Lipofectamine 3000 transfection reagent (Invitrogen) according to the manufacturer's instructions. Cells transfected with the empty vector were considered as the negative control (Ov‐NC). Plasmids were acquired from GenePharma company. At 48 h posttransfection, cells were collected for subsequent experiments.

### 2.4 Cell viability assay

After the indicated treatment, cell viability was tested by a cell counting kit‐8 (CCK‐8) kit obtained from Beyotime. THP‐1‐derived macrophages or HK‐2 cells were seeded into 96‐well plates (1 × 10^4^/well) and incubated for 24 h. Then, CCK‐8 solution was added to each well for an extra 2 h incubation at 37°C. The absorbance of the reaction product was quantified at 450 nm using a microplate reader (Bio‐Rad).

### 2.5 Measurement of inflammatory factors contents

Enzyme‐linked immunosorbent assay (ELISA) kits (Westang Biotechnology) were used to evaluate the concentrations of secreted TNF‐α, IL‐1β, and IL‐6 in the supernatant of cultured THP‐1‐derived macrophages confirmed to the recommendations of the manufacturer. The absorbance was detected using a microplate reader (Bio‐Rad).

### 2.6 Flow cytometry analysis

HK‐2 cells cultured with different CM from THP‐1‐derived macrophages were collected and centrifuged at 2000*g* for 3 min at room temperature. After cells were rinsed with the buffer solution three times, they were mixed with 5 µL Annexin‐V–fluorescein isothiocyanate (Vazyme) and 5 µL propidium iodide (Vazyme) for 15 min away from light. The ratio of apoptotic cells was determined by a flow cytometer (BD Biosciences).

### 2.7 Experimental animals

Male C57BL/6 mice (22–25 g, 6–8 weeks) were provided by Hangzhou Ziyuan Laboratory Animal Technology Co., Ltd. Mice were maintained in an environment with ambient temperature of 18–22°C, relative humidity of 50%–60%, and the absence of pathogens. All animals were given free access to standard rodent diet and drinking water. All experimental procedures were approved by the Animal Ethics Committee of the Second Affiliated Hospital of Wenzhou Medical University.

### 2.8 Establishment of sepsis‐induced kidney mice model

After 1 week of adaptive feeding, the mice were divided into three groups: control, sepsis, and sepsis + Deh groups. CLP was performed on mice to create a murine sepsis model, as previously described.^24^ Mice in the Deh treatment group were given 100 mg/kg of Deh for three consecutive days before CLP operation. Sodium pentobarbital (50 mg/kg) was used to anesthetize the mice. Then, a midline abdominal incision was made to expose the cecum and ligate the distal end of the cecal valve. Double punctures were made using a 21‐gauge needle and expelling a small amount of intestinal content into the abdominal cavity. Mice in the control group underwent laparotomy and bowel manipulation without ligation or perforation. All animals were monitored until killed with 150 mg/kg sodium pentobarbital by intraperitoneal injection 24 h after surgery. The blood and kidney tissues were collected for subsequent experiments.

### 2.9 Biochemical analyses in blood

Blood samples were centrifuged at 3000*g* for 10 min at 4°C for collecting serum. The concentrations of blood urea nitrogen (BUN) and serum creatinine (SCr) were examined by means of the corresponding assay kits (Nanjing Jiancheng Bioengineering Institute) in light of the manufacturer's guidance.

### 2.10 Renal histological assessment

Hematoxylin–eosin (H&E) staining was used to examine the histopathological changes of renal tissue. In detail, the renal tissues were fixed with 4% paraformaldehyde for 24 h, dehydrated, and embedded in paraffin. Then, 5 μm thickness paraffin‐embedded tissue sections were deparaffinized in xylene and rehydrated in graded ethanol. To analyze pathological changes, the sections were stained with hematoxylin for 10 min and then eosin for 5 min. The morphological examination was conducted and photos were taken under a light microscope (Olympus).

### 2.11 Immunofluorescence staining

The 5 μm thickness paraffin‐embedded tissue sections were deparaffinized in xylene and rehydrated in graded ethanol. Then, antigen retrieval was performed using sodium citrate buffer. THP‐1‐derived macrophages were fixed with 4% paraformaldehyde for 30 min at room temperature, followed by permeabilization with 0.1% Triton X‐100 for 20 min. After blocking with 5% bovine serum albumin (BSA), the slides were probed with inducible nitric‐oxide synthase (iNOS), Arginase‐1 (Arg‐1) or F4/80 antibodies at 4°C. The next day, Alexa Fluor‐488‐conjugated secondary antibody was adopted for incubation with the samples. The cell nuclei were stained by 4’,6‐diamidino‐2‐phenylindole (DAPI). The images were captured with a fluorescence microscope (Olympus).

### 2.12 Immunohistochemical analysis

For immunohistochemical analysis, the initial steps were the same as those for immunofluorescence staining of renal tissues until antigen repair. The sections were blocked with 5% BSA. After incubation with the primary antibody against iNOS (22226‐1‐AP, 1:200 dilution; Proteintech) and Arg‐1 (16001‐1‐AP, 1:200 dilution; Proteintech) overnight at 4°C, slides were probing with secondary antibody (sc‐2357, 1:100 dilution; Santa Cruz Biotechnology) at room temperature for 30 min. Subsequently, the slides were stained with diaminobenzidine and hematoxylin, followed by dehydrated and mounted. The images were observed and photographed under a light microscope (Olympus).

### 2.13 Terminal‐deoxynucleotidyl transferase‐mediated nick end labeling (TUNEL) staining

Apoptotic cells in renal tissues were detected with a TUNEL Apoptosis Assay Kit (YEASEN) according to the manufacturer's instructions. The sections were deparaffinized and rehydrated through xylene/ethanol, then treated with proteinase K (Beyotime) for 30 min at 37°C. Afterwards, the slides were incubated with TUNEL detection solution for 1 h. The cell nuclei were stained with DAPI and exhibited the blue light. The images were captured under a fluorescence microscope (Olympus).

### 2.14 Real‑time quantitative polymerase chain reaction (PCR)

Total RNA was extracted from cultured cells and renal tissues using TRIzol® reagent (Takara). the PrimeScript RT reagent kit (Takara) was used to synthesize the complementary DNA according to the instructions provided by the manufacturer. Target genes were amplified using SYBR Green PCR Master Mix Reagents (Takara) on the ABI 7500 Real‐Time PCR system (Applied Biosystems). The relative gene expression was determined with 2^−ΔΔCT^ method.^25^ Amplification of glyceraldehyde 3‐phosphate dehydrogenase (GAPDH) was used as an internal control.

### 2.15 Western blot analysis

Total proteins were isolated from cultured cells and renal tissues using RIPA lysis buffer (Beyotime), followed by quantification of protein concentration with a bicinchoninic acid protein assay kits (Beyotime). The protein samples were adjusted to the same amount (30 µg/lane) and subjected to 10% sodium dodecyl‐sulfate polyacrylamide gel electrophoresis, followed by electrophoretic transferring onto polyvinylidene difluoride membranes. Then, 5% skimmed milk was employed as the blocking reagent. Then, primary antibodies were added followed by incubation overnight at 4°C. After incubating with the goat anti‐rabbit horseradish peroxidase‐linked secondary antibody (7074P2, 1:10,000; Cell Signaling Technology) for 1 h at 37°C, immunoblots were visualized using ECL Detection Reagent (Millipore), and analyzed using Image J software. The expression of protein was normalized to GAPDH. Anti‐CD86 (13395‐1‐AP, 1:2000 dilution), anti‐iNOS (22226‐1‐AP, 1:1000 dilution), anti‐CD206 (18704‐1‐AP, 1:1000 dilution), anti‐Arg‐1 (16001‐1‐AP, 1:1000 dilution), and anti‐DUSP3 (28284‐1‐AP, 1:500 dilution) antibodies were purchased from Proteintech. Anti‐B‐cell lymphoma 2 (BCL2; 3498T, 1:1000 dilution), anti‐Bcl‐2‐associated X protein (Bax; 2772T, 1:1000 dilution), anti‐cleaved caspase3 (9661T, 1:1000 dilution), anti‐caspase3 (9662 S, 1:1000 dilution), and anti‐GAPDH (2118S, 1:1000 dilution) antibodies were obtained from Cell Signaling Technology.

### 2.16 Statistical analyses

The experimental data were presented as mean ± SD and analyzed by GraphPad Prism 8 software (GraphPad Software, Inc.). Statistical calculations of the data were performed using one‐way analysis of variance followed by Tukey's test. Statistical significance was set at *p* < .05.

## 3 RESULTS

### 3.1 Deh treatment alleviates the inflammatory factors secretion in THP‐1‐derived macrophages

**Figure 1:**
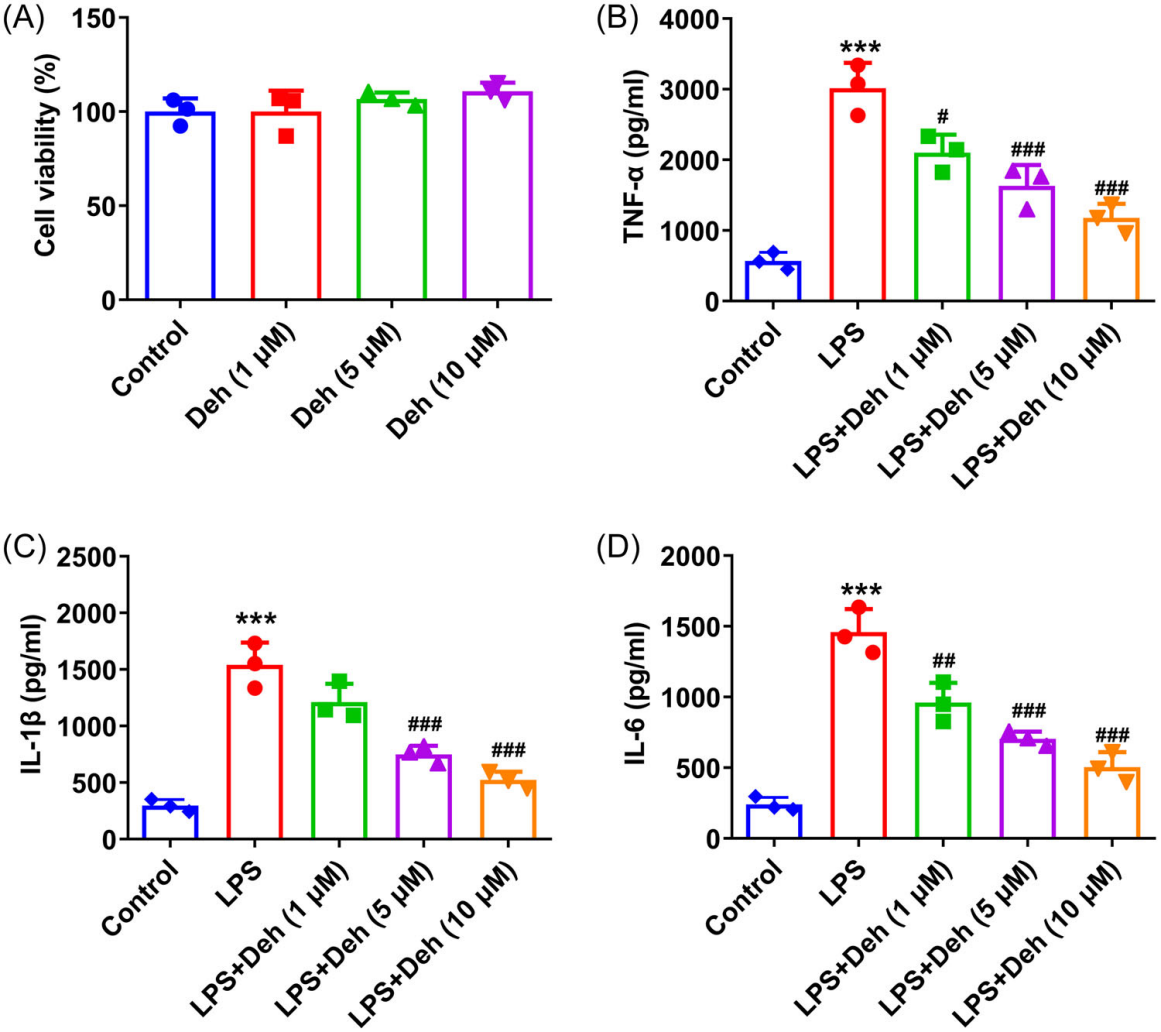
Dehydroandrographolide (Deh) treatment alleviated the inflammatory factors secretion in THP‐1‐derived macrophages. (A) The viability of THP‐1‐derived macrophages after treatment with various concentrations of Deh was assessed by cell counting kit‐8 (CCK‐8) assay. The levels of (B) tumor necrosis factor‐α (TNF‐α), (C) interleukin‐1β (IL‐1β), and (D) interleukin‐6 (IL‐6) in THP‐1‐derived macrophages with or without lipopolysaccharide (LPS) and Deh treatment were examined using enzyme‐linked immunosorbent assay kits. Data were displayed as mean ± SD. *N* = 3. Statistical analyses were performed using one‐way analysis of variance followed by Turkey's posthoc test. *p* < .05 was considered to indicate a statistically significant difference. ***p < .001 versus control group; ^#^ *p* < .05, ^##^ *p* < .01, ^###^ *p* < .001 versus LPS group.

As shown in Figure 1A, Deh treatment from 1 to 10 μM had no significant effect on the viability of THP‐1‐derived macrophages compared with the control group. Then, THP‐1‐derived macrophages were induced by LPS in the presence or absence of various concentrations of Deh. It could be found that LPS stimulation led to the significantly elevated TNF‐α, IL‐1β and IL‐6 contents when compared to the control group (Figure 1B–D). On the contrary, Deh treatment gradually reduced the levels of these inflammatory factors in THP‐1‐derived macrophages exposed to LPS. These results suggest that Deh attenuates the inflammatory factors secretion in THP‐1‐derived macrophages.

### 3.2 Deh treatment induces the polarization of M1 macrophages to M2 in THP‐1‐derived macrophages exposed to LPS

**Figure 2:**
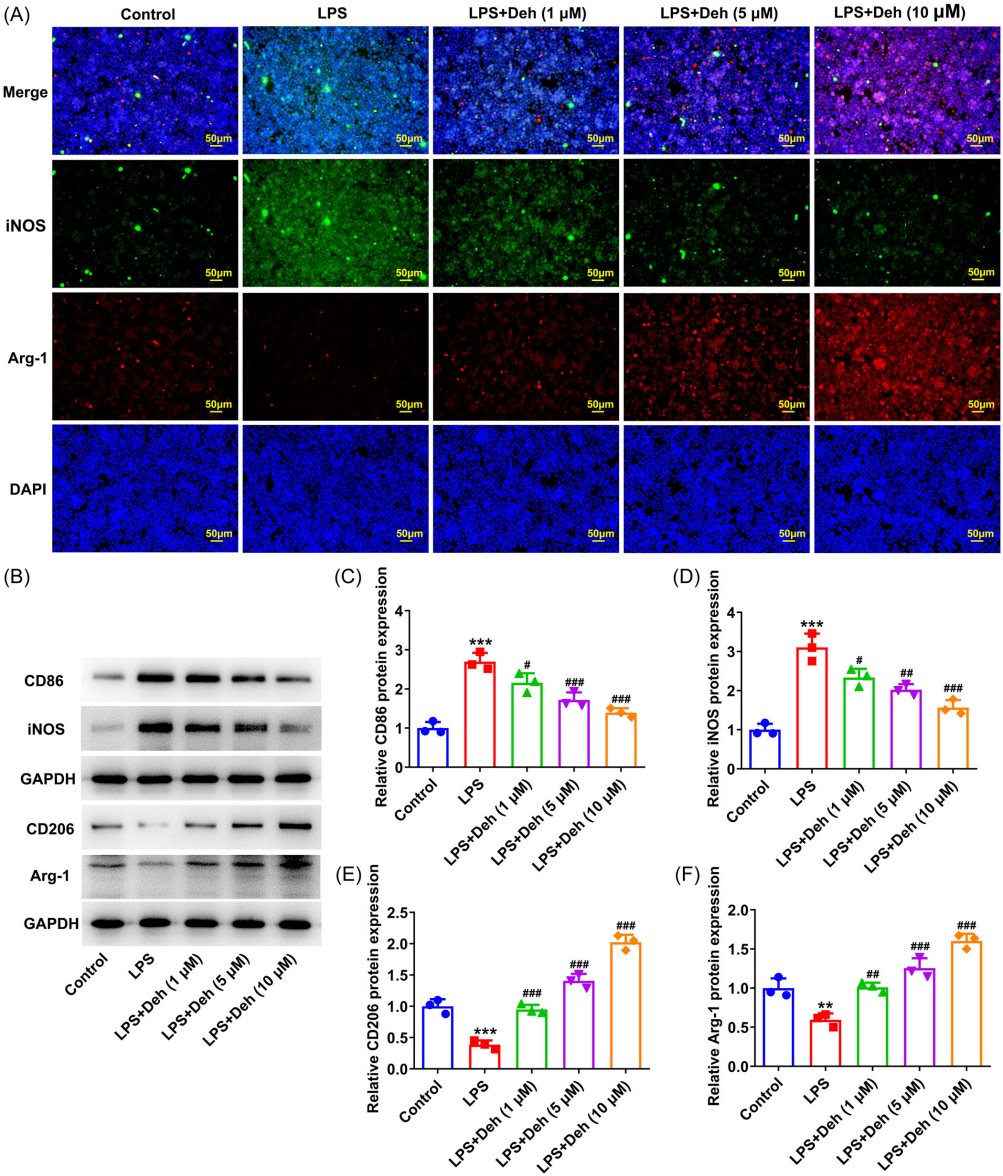
Dehydroandrographolide (Deh) treatment induced the polarization of M1 macrophages to M2 in THP‐1‐derived macrophages exposed to lipopolysaccharide (LPS). (A) The expression of inducible nitric‐oxide synthase (iNOS) and Arginase‐1 (Arg‐1) in THP‐1‐derived macrophages with or without LPS and Deh treatment was evaluated by immunofluorescence staining. (B) Representative images of CD86, iNOS, CD206, and Arg‐1 expression in western blot assay in THP‐1‐derived macrophages with or without LPS and Deh treatment. (C–F) Quantification of CD86, iNOS, CD206, and Arg‐1 expression in western blot assay. Data were displayed as mean ± SD. *N* = 3. Statistical analyses were performed using one‐way analysis of variance followed by Turkey's posthoc test. *p* < .05 was considered to indicate a statistically significant difference. **p < .01, ***p < .001 versus control group; ^#^ *p* < .05, ^##^ *p* < .01, ^###^ *p* < .001 versus LPS group. GAPDH, glyceraldehyde 3‐phosphate dehydrogenase; DAPI, 4’,6‐diamidino‐2‐phenylindole.

Then, the expression of markers related to M1 phenotype and M2 phenotype macrophages was detected by using immunofluorescence staining and western blot. As exhibited in Figure 2A, notably increased iNOS and decreased Arg‐1 fluorescence intensity were observed in THP‐1‐derived macrophages in the LPS group as comparison to the control group, which were reversed by Deh stimulation in a dose‐dependent manner. Consistently, when compared to the control group, LPS induced the upregulated expression of CD86 and iNOS (M1 phenotype macrophages markers), as well as the downregulated expression of CD206 and Arg‐1 (M2 phenotype macrophages markers) (Figure 2B–F). Deh treatment alleviated the impacts of LPS exposure on the expression of aforementioned proteins. Above data reveal that Deh treatment induces the polarization of M1 macrophages to M2 in THP‐1‐derived macrophages exposed to LPS.

### 3.3 Deh treatment downregulates DUSP3 expression in THP‐1‐derived macrophages exposed to LPS

**Figure 3:**
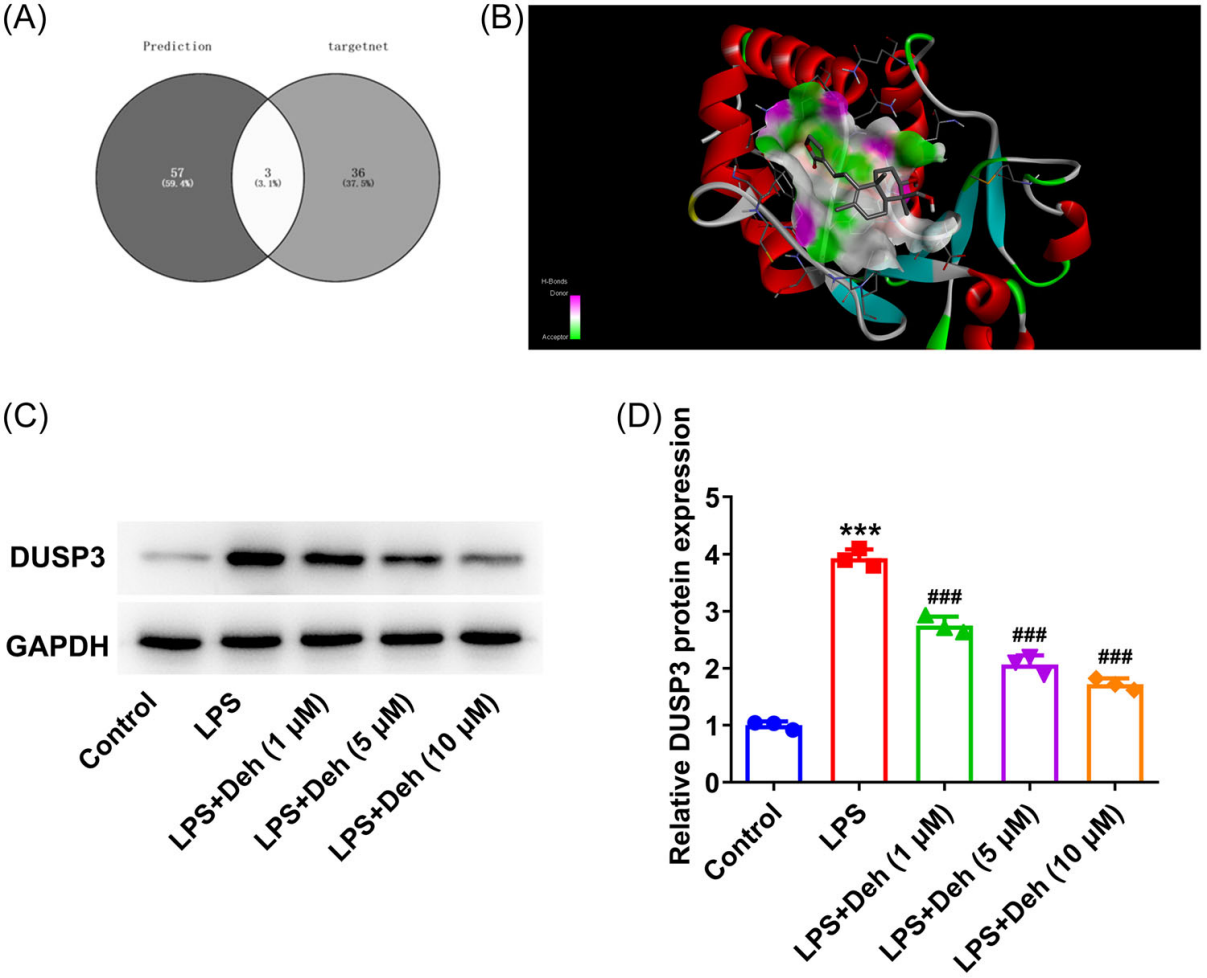
Dehydroandrographolide (Deh) treatment downregulates dual specificity phosphatase 3 (DUSP3) expression in THP‐1‐derived macrophages exposed to lipopolysaccharide (LPS). (A) The potential target genes of Deh were predicted by Prediction and Targetnet databases, and the results were exhibited using Venn diagram. (B) Result of molecular docking between Deh and DUSP3. (C) Representative image of DUSP3 expression in western blot assay in THP‐1‐derived macrophages with or without LPS and Deh treatment. (D) Quantification of DUSP3 expression in western blot assay. Data were displayed as mean ± SD. *N* = 3. Statistical analyses were performed using one‐way analysis of variance followed by Turkey's posthoc test. *p* < .05 was considered to indicate a statistically significant difference. ***p < .001 versus control group; ^###^ *p* < .001 versus LPS group. GAPDH, glyceraldehyde 3‐phosphate dehydrogenase.

To study the underlying mechanism of Deh in the regulation of LPS‐induced inflammation of THP‐1‐derived macrophages, the potential target genes of Deh were predicted by Prediction and Targetnet databases, and the results were exhibited using Venn diagram (Figure 3A). It was found that DUSP3, AR, and CHEM5 were three genes that could potentially been targeted by Deh. Among them, DUSP3 was found to bind to Deh by molecular docking (Figure 3B). The further western blot assay indicated that LPS induced upregulation of DUSP3 in THP‐1‐derived macrophages was reversed by the addition of Deh in a dose‐dependent manner (Figure 3C,D). Overall, Deh treatment downregulates DUSP3 expression in THP‐1‐derived macrophages exposed to LPS.

### 3.4 DUSP3 overexpression attenuates the impacts of Deh on the inflammation of LPS‐induced THP‐1‐derived macrophages and the polarization of M1 macrophages to M2

**Figure 4:**
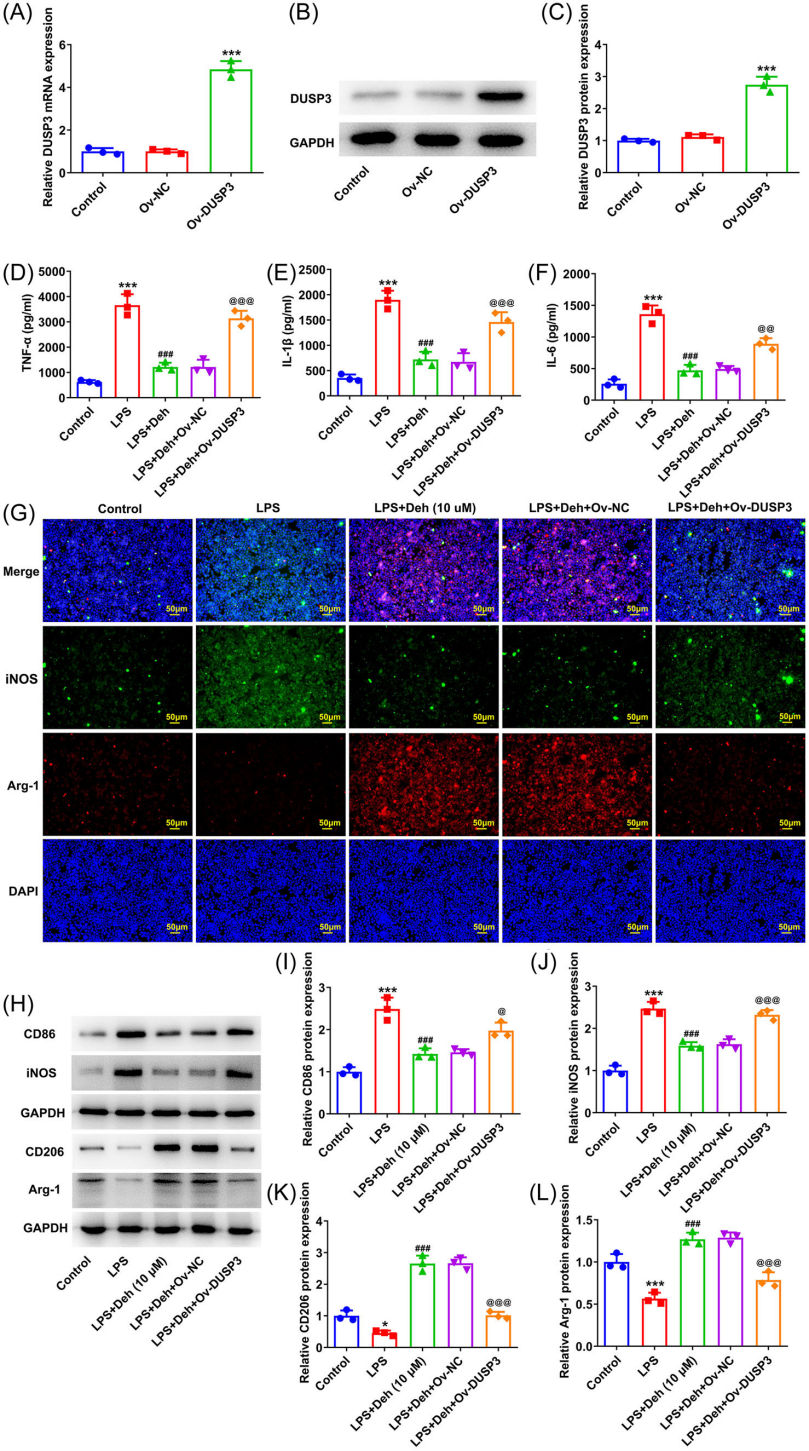
Dual specificity phosphatase 3 (DUSP3) overexpression attenuated the impacts of dehydroandrographolide (Deh) on the inflammation of lipopolysaccharide (LPS)‐induced THP‐1‐derived macrophages and the polarization of M1 macrophages to M2. (A) Real‑time quantitative polymerase chain reaction (RT‐qPCR) and (B, C) western blot analysis were employed to detect the transfection efficiency of Ov‐DUSP3 in THP‐1‐derived macrophages. Data were displayed as mean ± SD. *N* = 3. Statistical analyses were performed using one‐way analysis of variance followed by Turkey's posthoc test. *p* < .05 was considered to indicate a statistically significant difference. **p < .01, ***p < .001 versus Ov‐NC group. Assessment of (D) tumor necrosis factor‐α (TNF‐α), (E) interleukin‐1β (IL‐1β), and (F) interleukin‐6 (IL‐6) levels using enzyme‐linked immunosorbent assay kits. (G) Immunofluorescence staining detected inducible nitric‐oxide synthase (iNOS) and Arginase‐1 (Arg‐1) expression. (H) Representative images of CD86, iNOS, CD206, and Arg‐1 expression in western blot assay. (I–L) Quantification of CD86, iNOS, CD206, and Arg‐1 expression in western blot assay. Data were displayed as mean ± SD. *N* = 3. Statistical analyses were performed using one‐way analysis of variance followed by Turkey's posthoc test. *p* < .05 was considered to indicate a statistically significant difference. ***p < .001 versus control group; ^###^ *p* < .001 versus LPS group; ^@^ *p* < .05, *p* ^@@^ < .01, *p* ^@@@^ < .001 versus LPS + Deh + Ov‐NC group. GAPDH, glyceraldehyde 3‐phosphate dehydrogenase; mRNA, messenger RNA.

Subsequently, DUSP3 was overexpressed by transfection with Ov‐DUSP3 plasmid. As presented in Figure 4A–C, compared with the empty vector group, DUSP3 expression was remarkably upregulated in THP‐1‐derived macrophages after transfection with Ov‐DUSP3. Then, results of ELISA revealed that DUSP3 overexpression dramatically elevated the concentrations of TNF‐α, IL‐1β, and IL‐6 in THP‐1‐derived macrophages when compared to the LPS + Deh + Ov‐NC group (Figure 4D–F). In addition, the iNOS fluorescence intensity was enhanced while Arg‐1 fluorescence intensity was attenuated after transfection Ov‐DUSP3 into THP‐1‐derived macrophages with LPS and Deh exposure (Figure 4G). Consistently, compared with the LPS+Deh+Ov‐NC group, DUSP3 overexpression alleviated the polarization of M1 macrophages to M2, as evidenced by the upregulated CD86 and iNOS expression, as well as the downregulated CD206 and Arg‐1 expression (Figure 4H–L). Above results confirm that Deh relieves the inflammation of LPS‐induced THP‐1‐derived macrophages and induces the polarization of M1 macrophages to M2 by downregulating DUSP3.

### 3.5 Deh alleviates the apoptosis and inflammation of renal tubular epithelial cells by inducing the polarization of M1 macrophages to M2 in THP‐1‐derived macrophages through downregulating DUSP3

**Figure 5:**
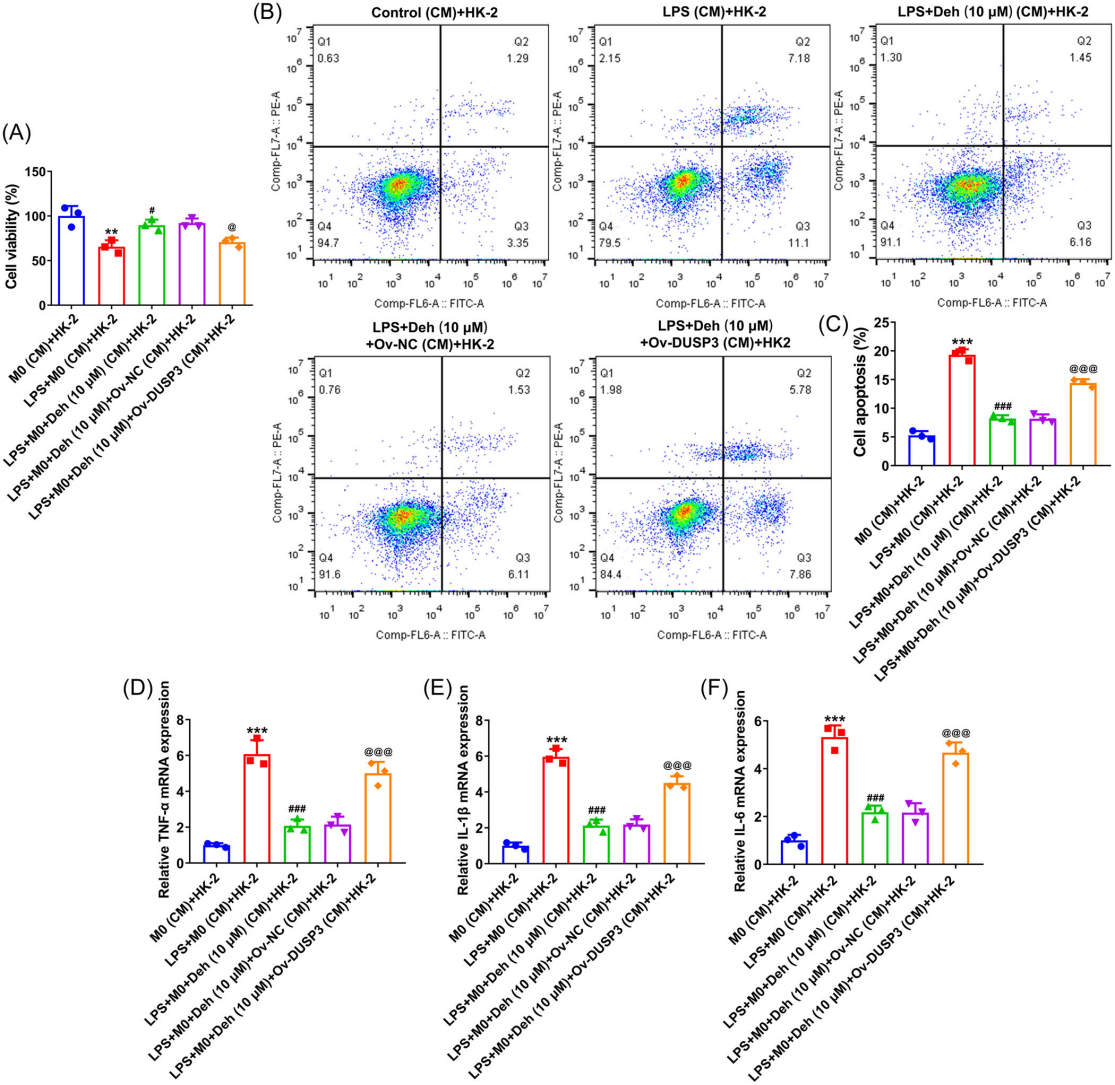
Dehydroandrographolide (Deh) alleviated the apoptosis and inflammation of renal tubular epithelial cells by inducing the polarization of M1 macrophages to M2 in THP‐1‐derived macrophages through downregulating dual specificity phosphatase 3 (DUSP3). (A) The viability of HK‐2 cells after cultured in different conditioned medium from THP‐1‐derived macrophages was examined by cell counting kit‐8 (CCK‐8) assay. (B) Flow cytometry analysis was used to assess the apoptosis of HK‐2 cells. (C) Quantification of cell apoptosis rate in flow cytometry analysis. Real‑time quantitative polymerase chain reaction (RT‐qPCR) was adopted for the detection of (D) tumor necrosis factor‐α (TNF‐α) (E) interleukin‐1β (IL‐1β) and (F) interleukin‐6 (IL‐6) levels in HK‐2 cells. Data were displayed as mean ±SD. *N* = 3. Statistical analyses were performed using one‐way analysis of variance (ANOVA) followed by Turkey's posthoc test. *p* < .05 was considered to indicate a statistically significant difference. **p < .01, ***p < .001 versus control (condition medium, CM) group; ^#^ *p* < .05, ^###^ *p* < .001 versus lipopolysaccharide (LPS) (CM) + HK‐2 group; ^@^ *p* < .05, *p* ^@@@^ < .001 versus LPS + Deh (10 μM)+Ov‐NC (CM) + HK‐2 group.

In this section, the effects of Deh on renal tubular epithelial cells (HK‐2) were studied by using conditioned medium to culture HK‐2 cells. As what is observable from Figure 5A, the viability of HK‐2 cells was significantly decreased after incubation in LPS (CM) compared with that in the control (CM). The further Deh treatment increased HK‐2 cell viability. Of note, HK‐2 cells cultured in the conditioned medium from LPS + Deh (10 μM) + Ov‐DUSP3 showed the reduced viability relative to the LPS + Deh (10 μM) + Ov‐NC (CM) + HK‐2 group. Besides, LPS stimulation notably elevated the apoptosis ratio of HK‐2 cells, which was restored by Deh administration (Figure 5B,C). By contrast, DUSP3 overexpression reversed the inhibitory effect of Deh on the apoptosis of HK‐2 cells. Additionally, significantly increased expression levels of TNF‐α, IL‐1β and IL‐6 were observed in HK‐2 cells cultured in LPS conditioned medium (Figure 5D–F). However, HK‐2 cells in the CM from THP‐1‐derived macrophages treated with LPS and Deh displayed decreased TNF‐α, IL‐1β and IL‐6 levels, the further Ov‐DUSP3 transfection alleviated this effect. Above data provide evidence that Deh alleviates the apoptosis and inflammation of HK‐2 cells by inducing the polarization of M1 macrophages to M2 in THP‐1‐derived macrophages through downregulating DUSP3.

### 3.6 Deh improves the renal function, ameliorates pathological injury and downregulates DUSP3 expression in sepsis‐induced mice

**Figure 6:**
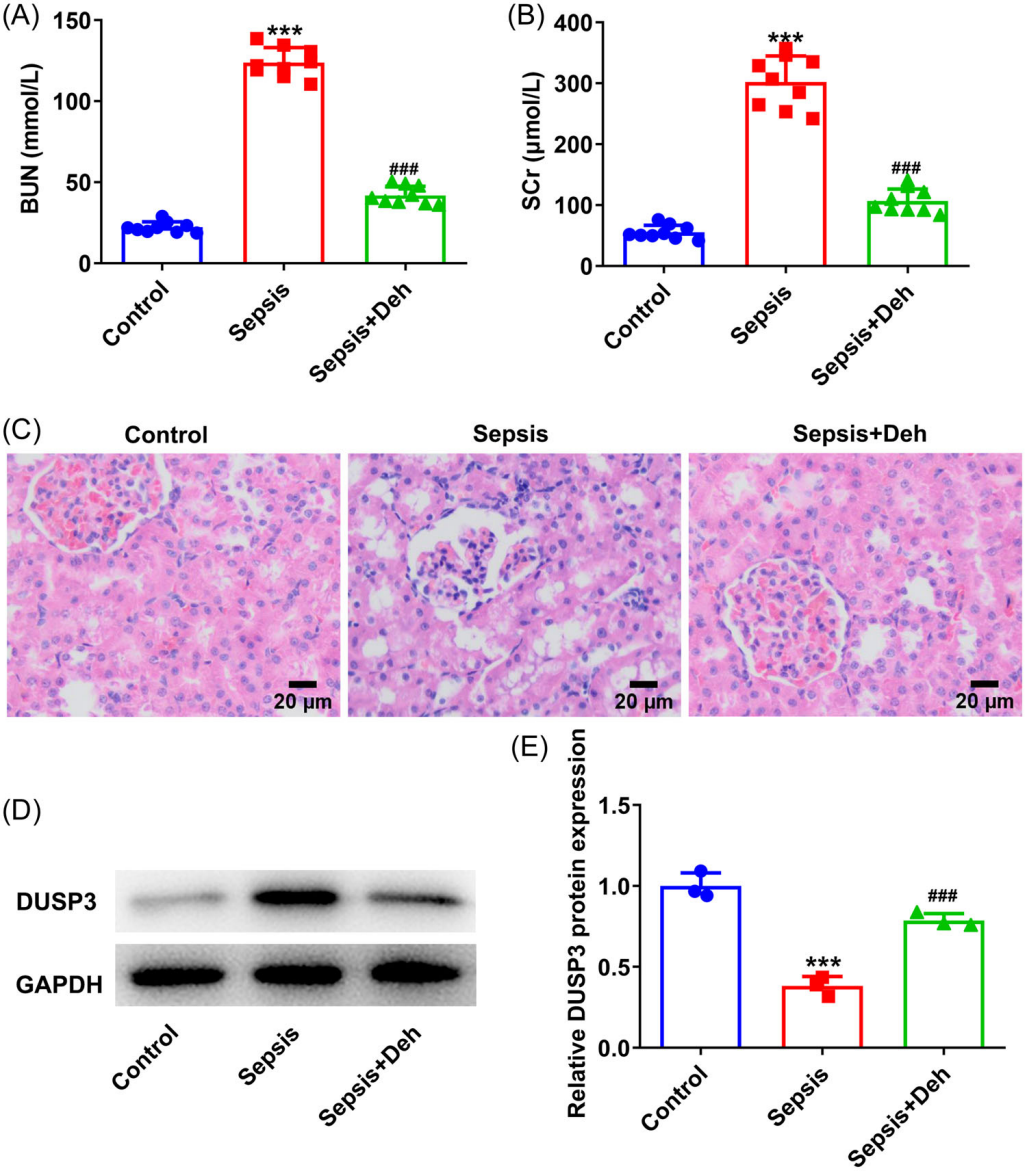
Dehydroandrographolide (Deh) improved the renal function, ameliorated pathological injury and downregulated dual specificity phosphatase 3 (DUSP3) expression in sepsis‐induced mice. The levels of (A) blood urea nitrogen (BUN) and (B) serum creatinine (SCr) were detected by the commercial available kits. Data were displayed as mean ± SD. *N* = 9. Statistical analyses were performed using one‐way analysis of variance (ANOVA) followed by Turkey's posthoc test. *p* < .05 was considered to indicate a statistically significant difference. **p < .001 versus control group; ^###^ *p* < .001 versus sepsis group. (C) The histopathological changes of renal tissues were evaluated by hematoxylin–eosin (H&E) staining. (D) Western blot analysis was used to assess DUSP3 expression in renal tissues. (E) Quantification of DUSP3 expression in western blot assay. Data were displayed as mean ± SD. *N* = 3. Statistical analyses were performed using one‐way ANOVA followed by Turkey's posthoc test. *p* < .05 was considered to indicate a statistically significant difference. ***p < .001 versus control group; ^###^ *p* < .001 versus sepsis group.

To further analyze the protective effects of Deh on sepsis‐associated AKI, a CLP‐induced sepsis mice model was established. It was found that the levels of renal function markers BUN and SCr were higher than that in the control group (Figure 6A,B). The further Deh administration significantly decreased both BUN and SCr concentrations. Results of H&E staining revealed that Deh treatment effectively alleviated kidney pathological damage characterized by disordered glomerular structure, edematous with larger cellular volume and vacuolar degeneration induced by sepsis (Figure 6C). Particularly, DUSP3 expression was conspicuously upregulated in the renal tissues of mice in the sepsis group, and Deh addition downregulated DUSP3 expression (Figure 6D,E). These data demonstrate that Deh protects against sepsis‐induced renal injury and downregulates DUSP3 expression in renal tissues.

### 3.7 Deh induces the polarization of M1 macrophages to M2 in sepsis‐induced AKI mice

**Figure 7:**
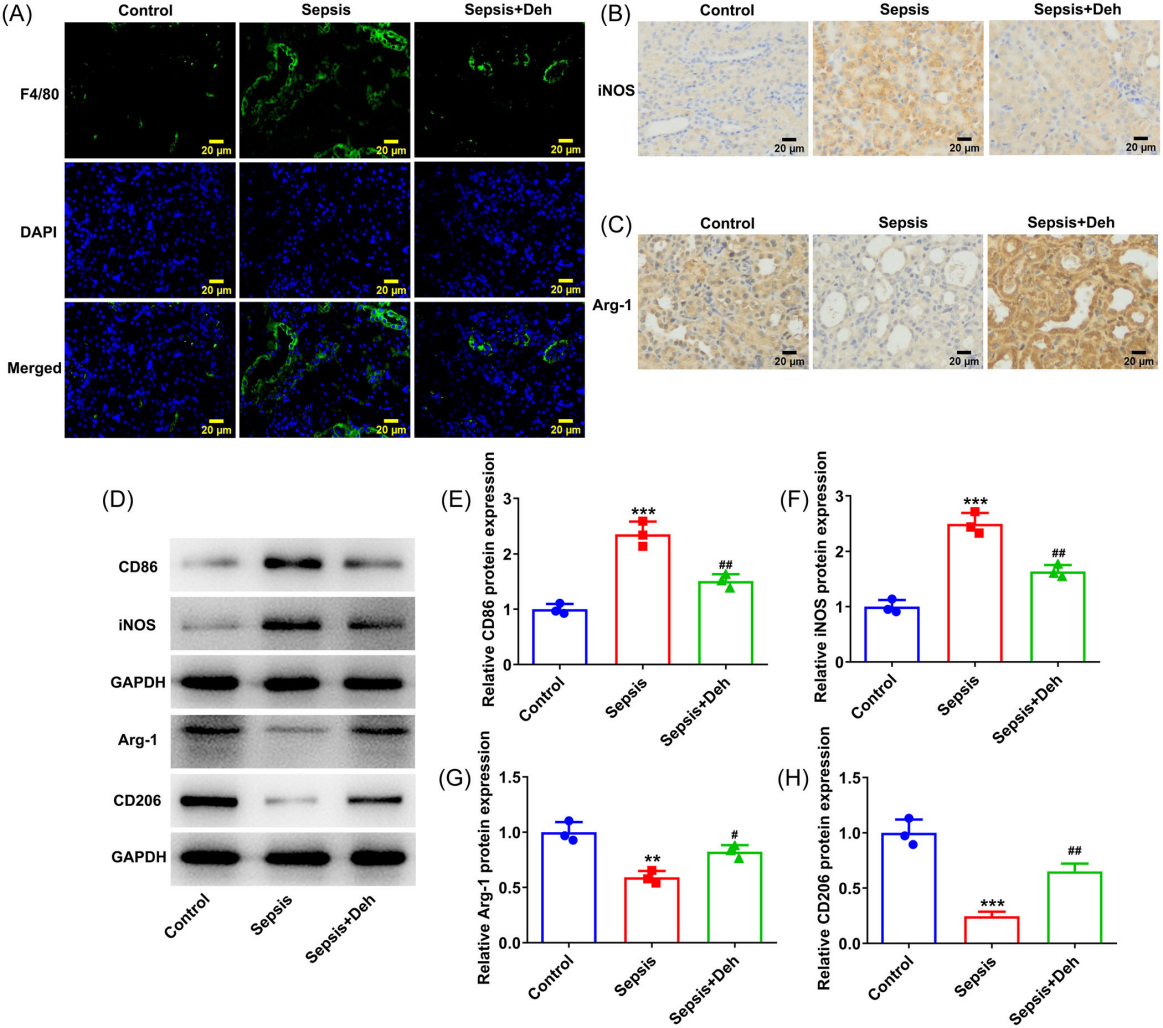
Dehydroandrographolide (Deh) induced the polarization of M1 macrophages to M2 in sepsis‐induced acute kidney injury (AKI) mice. (A) Immunofluorescence staining detected F4/80 expression in renal tissues. The levels of (B) inducible nitric‐oxide synthase (iNOS) and (C) Arginase‐1 (Arg‐1) were examined by immunohistochemical analysis. (D) Representative images of CD86, iNOS, CD206, and Arg‐1 expression in renal tissues in western blot assay. (E–H) Quantification of CD86, iNOS, CD206, and Arg‐1 expression in western blot analysis assay. Data were displayed as mean ± SD. *N* = 3. Statistical analyses were performed using one‐way analysis of variance followed by Turkey's posthoc test. *p* < .05 was considered to indicate a statistically significant difference. **p < .01, ***p < .001 versus control group; ^#^ *p* < .05, ^##^ *p* < .01 versus sepsis group. GAPDH, glyceraldehyde 3‐phosphate dehydrogenase.

The subsequently experiments suggested that sepsis caused upregulated expression of F4/80, iNOS and CD86, as well as downregulated expression of Arg‐1 and CD206 in renal tissues of mice were reversed by Deh treatment (Figure 7A–H). To summarize, Deh induces the polarization of M1 macrophages to M2 in sepsis‐induced AKI mice.

### 3.8 Deh suppresses inflammation and apoptosis of renal tissues in sepsis‐induced AKI mice

**Figure 8:**
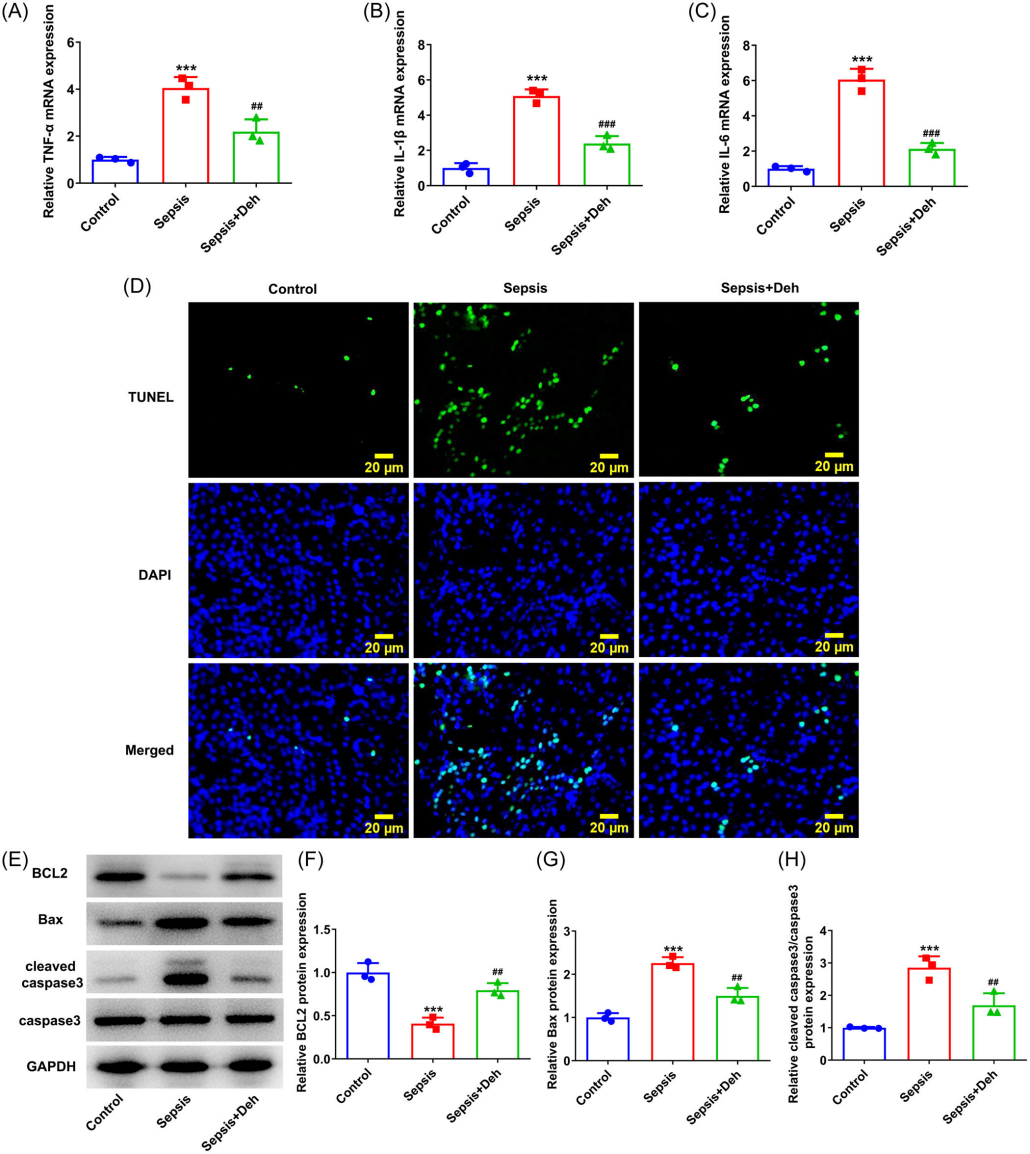
Dehydroandrographolide (Deh) suppressed inflammation and apoptosis of renal tissues in sepsis‐induced acute kidney injury (AKI) mice. The messenger RNA (mRNA) expression of (A) tumor necrosis factor‐α (TNF‐α) (B) interleukin‐1β (IL‐1β), and (C) interleukin‐6 (IL‐6) was tested by Real‑time quantitative polymerase chain reaction (RT‐qPCR). (D) Apoptosis of renal tissues cells was tested by terminal‐deoxynucleotidyl transferase‐mediated nick end labeling (TUNEL) staining. (E) Representative images of B‐cell lymphoma 2 (BCL2), Bcl‐2‐associated X protein (Bax), cleaved caspase3, and caspase3 expression in renal tissues in western blot analysis assay. (F–H) Quantification of BCL2, Bax, cleaved caspase3, and caspase3 expression in western blot analysis assay. Data were displayed as mean ± SD. *N* = 3. Statistical analyses were performed using one‐way analysis of variance followed by Turkey's posthoc test. *p* < .05 was considered to indicate a statistically significant difference. ***p < .001 versus control group; ^##^ *p* < .01, ^###^ *p* < .001 versus sepsis group. GAPDH, glyceraldehyde 3‐phosphate dehydrogenase.

As exhibited in Figure 8A–C, CLP operation led to the elevated TNF‐α, IL‐1β and IL‐6 expression in renal tissues relative to that in the control group, which were remarkably reduced following Deh treatment. Moreover, the apoptosis of renal tissues was remarkably increased in the sepsis group as compassion to the control group (Figure 8D). Of note, Deh treatment decreased the apoptosis level of renal tissues when compared with the sepsis group. Consistently, the downregulated BCL2 expression and upregulated Bax and cleaved capase3 expression induced by sepsis was restored by Deh administration (Figure 8E–H). These results support that Deh suppresses inflammation and apoptosis of renal tissues in sepsis‐induced AKI mice.

## 4 DISCUSSION

Sepsis is perceived as lethal tissue damage and organ dysfunction. As a serious and common complication of sepsis, AKI is a risk factor for progression to chronic kidney disease.^26^ Recently, clinical studies have suggested that AKI is associated with increased mortality in patients with sepsis.^27^, ^28^ In this study, we demonstrated that Deh, a main active constituent of *A. paniculata* (Burm. f) Nees, could protect against sepsis‐associated AKI in vitro and in vivo by facilitating M2 macrophage polarization by downregulating DUSP3.

Macrophages are involved in the process of the innate immune response and play a crucial role in sepsis and kidney disease processes.^29^, ^30^ An increasing number of research have validated that during sepsis, the concentration of cytokines and chemokines in the blood increases dramatically, resulting in toxic effects on tubular cells when they are concentrated into the ultrafiltrate on the surface of the tubular lumen.^31^ That is to say, septic AKI is closely related to the immune system, especially M2 macrophages. As alternatively activated macrophage, M2 macrophages can not only promote the regeneration and restrain the apoptosis of renal tubular cells, but also indirectly alleviate kidney injury by decreasing the inflammatory infiltrates in the renal interstitium.^32^, ^33^, ^34^ More importantly, M2 macrophages can defend the pro‐inflammatory function of M1 macrophages, thereby reducing the damage to the kidney caused by M1‐induced inflammation.^30^ Therefore, inducing macrophage polarization from M1 to M2 phenotype is closely related to the delay or even reversal of kidney injury, and it is of great value to apply its characteristics to clinical diseases and shorten the course of kidney disease in patients. Classical markers of M1 macrophages include CD86 and iNOS, and markers of M2 macrophages include CD206, Arg‐1, and F4/80.^35^, ^36^ Intriguingly, Deh has been reported to enhance innate immunity of intestinal tract.^16^ Deh decreased the levels of TNF‐α and IL‐6 in bile duct‐ligated mice or LPS‐induced primary Kupffer cells, thereby defending inflammatory activities in cholestatic liver injury.^17^ Of note, 3‐Deh protects against LPS‐induced inflammation in murine macrophage RAW 264.7 cells through the cholinergic anti‐inflammatory pathway.^37^ This study for the first time demonstrated that Deh attenuated the inflammation of renal tubular epithelial cells by inducing the polarization of M1 macrophages to M2 in THP‐1‐derived macrophages, which were also supported by the in vivo experiments of CLP‐induced mice with Deh treatment.

DUSP3, a small dual‐specificity protein phosphatase, locates on chromosome 17q21 in humans.^38^ DUSP3 is highly expressed monocytes and macrophages, and plays a significant role in the innate immune response.^39^ The anti‐inflammation effect of DUSP3 deficiency has been widely reported by researchers. For instance, DUSP3 loss‐of‐function relieves acute myocardial infarction damage through suppressing inflammation and apoptosis.^40^ DUSP3 silencing reduces kidney injury and inflammation after ischemia/reperfusion injury in mice.^20^ In the research on sepsis, it was found that DUSP3 deficiency protects mice from endotoxemia and multimicrobially induced septic shock.^19^ Particularly, DUSP3‐knockout mice display an increase in the proportion of M2‐type macrophages and a decrease in the expression of inflammatory factor TNF‐α.^18^ DUSP3 was predicted potentially been targeted by Deh by using bioinformatics tools and molecular docking in this study. We also demonstrated that Deh downregulated DUSP3 expression in both LPS‐induced THP‐1‐derived macrophages and kidney tissues of septic mice. The further DUSP3 overexpression alleviated the impacts of Deh on the induction of THP‐1‐derived macrophages polarization from M1 phenotype to M2 phenotype. Additionally, HK‐2 cells in the CM from DUSP3‐overexpressed THP‐1‐derived macrophages treated with LPS and Deh displayed the decreased viability and increased apoptosis and inflammation. Above results together suggested that Deh could protect against sepsis‐associated AKI in vitro and in vivo by facilitating M2 macrophage polarization by downregulating DUSP3.

Several limitations should be noted in this study. First, we only assessed the early therapeutic effects of Deh in sepsis‐associated AKI in vitro and in vivo, and we did not conduct any long‐term observations. Second, we only overexpressed DUSP3 to explore the regulatory effect of Deh on DUSP3, and the data about DUSP3 silencing did not included. Furthermore, it was found that *DUSP3*, *AR*, and *CHEM5* were three genes that could potentially been targeted by Deh (Figure 3A). It is worthwhile to investigate whether Deh can attenuate the progression of sepsis‐associated AKI by early regulation of AR and CHEM5.

To be concluded, the present study elucidated that Deh exerted an alleviating effect on septic AKI, likely resulting from the promotion of M2 macrophage polarization by downregulating DUSP3. These findings provide a new understanding of the pathological mechanism of septic AKI and a theoretical basis for the use of Deh in the prevention and treatment of this disease.

## AUTHOR CONTRIBUTIONS

Hailun Cai designed the research study. Yanyan Shao, Weihao Yu, and Hailun Cai performed the research. Yanyan Shao and Weihao Yu made substantial contributions to the analysis and interpretation of data. Yanyan Shao wrote the paper and Hailun Cai revised it. All authors have read and approved the final manuscript.

## CONFLICT OF INTEREST STATEMENT

The authors declare no conflict of interest.

## ETHICS STATEMENT

All animal studies were approved by the Animal Ethics Committee of the Second Affiliated Hospital of Wenzhou Medical University.

## Data Availability

Enquiries about data availability should be directed to the corresponding author.
